# Re-sequencing and transcriptome analysis reveal rich DNA variations and differential expressions of fertility-related genes in neo-tetraploid rice

**DOI:** 10.1371/journal.pone.0214953

**Published:** 2019-04-05

**Authors:** Xuejun Bei, Muhammad Qasim Shahid, Jinwen Wu, Zhixiong Chen, Lan Wang, Xiangdong Liu

**Affiliations:** 1 State Key Laboratory for Conservation and Utilization of Subtropical Agro-Bioresources, South China Agricultural University, Guangzhou, China; 2 Guangdong Provincial Key Laboratory of Plant Molecular Breeding, South China Agricultural University, Guangzhou, China; 3 College of Agriculture, South China Agricultural University, Guangzhou, China; Nigde Omer Halisdemir University, TURKEY

## Abstract

Autotetraploid rice is a useful germplasm for polyploid rice breeding, however, low seed setting is the major barrier in commercial utilization of autotetraploid rice. Our research group has developed neo-tetraploid rice lines, which have the characteristics of high fertility and heterosis when crossed with autotetraploid rice. In the present study, re-sequencing and RNA-seq were employed to detect global DNA variations and differentially expressed genes (DEGs) during meiosis stage in three neo-tetraploid rice lines compared to their parents, respectively. Here, a total of 4109881 SNPs and 640592 InDels were detected in neo-tetraploid lines compared to the reference genome, and 1805 specific presence/absence variations (PAVs) were detected in three lines. Approximately 12% and 0.5% of the total SNPs and InDels identified in three lines were located in genic regions, respectively. A total of 28 genes, harboring at least one of the large-effect SNP and/or InDel which affect the integrity of the encoded protein, were identified in the three lines. Together, 324 specific mutation genes, including 52 meiosis-related genes and 8 epigenetics-related genes were detected in neo-tetraploid rice compared to their parents. Of these 324 genes, five meiosis-related and three epigenetics-related genes displayed differential expressions during meiosis stage. Notably, 498 specific transcripts, 48 differentially expressed transposons and 245 differentially expressed ncRNAs were also detected in neo-tetraploid rice. Our results suggested that genomic structural reprogramming, DNA variations and differential expressions of some important meiosis and epigenetics related genes might be associated with high fertility in neo-tetraploid rice.

## Introduction

Polyploidy plays an important role in plant evolution and could be an important source for plant breeders in future [1,2]. Over 70% of all angiosperm species have experienced whole genome duplication during the evolutionary process [3,4,5]. Polyploidy offers many advantages over diploid progenitors, such as increased variations in the expressions of dosage-regulated genes that evolved new biological functions, the largest vegetative organs, longer panicles, and high levels of heterosis [6,7,8,9,10].

Autotetraploid rice is a useful germplasm resource obtained by colchicine treatment, which showed higher genetic variation, greater ability of resistance against abiotic and biotic stresses, and higher biomass production than diploid rice [10, 11,12]. Intersubspecific hybrids (*indica* × *japonica*) of autotetraploid rice have a powerful biological and yield potential, and it is expected to become a new way to breed rice in the future [13]. However, low seed setting is the major barrier in commercial utilization of polyploid rice [14, 15]. Partial pollen sterility is one of the most important reasons for low fertility in autotetraploid rice, which caused by different factors, such as abnormal microtubule distribution pattern and chromosome behavior [16,17,18]. These abnormalities might be caused by the abrupt changes in the expression patterns of genes and miRNAs associated with meiosis [2, 19, 20]. Polyploidy could increase the interactions between pollen sterility loci and cause high meiosis abnormalities that lead to high pollen sterility in autotetraploid rice hybrids [18]. Another study revealed that pollen sterility mechanism is very complex and sequence variation, differential levels of methylation and differentially expressed genes have a strong influence on the fertility of autotetraploid rice [21]. Recently, the breeding procedure of neo-tetraploid rice that could overcome the sterility of autotetraploid rice hybrids has been reported. Moreover, they also employed transcriptome analysis of the neo-tetraploid rice and their hybrids to reveal differential expression patterns of genes associated with fertility [13].

Technological advances allow sequencing to be performed more economically and efficiently than ever before, and providing excellent opportunities to investigate biological problems. Re-sequencing technology had been utilized successfully in diploid rice and revealed huge genome-wide DNA variations involved in various agronomic traits [22, 23, 24, 25, 26]. However, little is known about DNA genome structural variations and gene expression in neo-tetraploid rice compared to autotetraploid parents. Therefore, we performed whole genome re-sequencing to detect the DNA genome wide variations between neo-tetraploid rice and their autotetraploid parents in the present study. Meanwhile, mRNA-seq was also employed to identify differentially expressed genes during meiosis, and to detect genes that might be associated with high seed setting in neo-tetraploid rice. The results of this study may help to explain the molecular mechanism of high fertility in neo-tetraploid rice.

## Materials and methods

### Ethics statement

No specific permissions were required for these locations/activities because all cultivars/lines were grown at our research station (farm of South China Agricultural University). We are doing research on these cultivars from more than two decades and our research group has generated these lines by crossing. We also confirmed that the field studies did not involve any endangered or protected species.

### Evaluation of agronomic traits and data analysis

Neo-tetraploid rice lines, 134 and 66, were developed in 2014, and named as Huaduo 4 and Huaduo 5 in 2017, respectively. Huaduo 4 and Huaduo 5 are sister-lines of Huaduo 3 (H3), which were developed by the same parents, Jackson-4x (maternal, T45) and 96025 (paternal, T44). A total of 20 plants from each autotetraploid rice parent (T44 and T45) and three neo-tetraploid rice lines were harvested from the field at maturity. Agronomic traits, including plant height, effective number of panicles per plant, filled grains per plant, empty grains per plant, total grains per plant, and grain yield per plant, were measured. These traits were selected from the Descriptors and Data standard for Rice (*Oryza sativa* L.) to describe the genetic variations between autotetraploid rice parents and three neo-tetraploid rice lines [27]. The single factor variance analysis of each trait (different combinations) was done by SPSS 16.0. Multiple comparison was done by Duncan's New Multiple-Range test (DMRT), using α = 0.05 significance level.

### Classification of chromosome behavior

Spikelets were collected from the rice plants with -2 to 2 cm between their flag leaf cushion and the second to last leaf cushion and fixed in Carnoy solution (ethanol: acetic acid [3:1, v/v] for at least 24 h. The samples were washed three times using 50% (v/v) ethanol and then stored in 70% (v/v) ethanol at 4°C. Anthers were removed from the floret using forceps and a dissecting needle and placed in a drop of 1% (w/v) acetocarmine on a glass slide. After 3 to 5 min, the glass slide was covered with a coverslip and examined under a microscope (Motic BA200). Meiotic stages were classified according to Wu et al. (2014) [19].

### Investigation of pollen and embryo sac fertility and seed setting

Five mature spikelets were collected from each line and all of them were fixed in Carnoy solution for 24 h to investigate the pollen fertility, and Potassium Iodide solution (I_2_-KI, 1%) was used to stain the pollen grain, which was observed under microscope. Pollen fertility was divided into four categories based on the color and morphology of pollen grain, i.e., normal fertile pollen, stained abortive, spherical abortive and typical abortive pollens [28]. WE-CLSM was used to observe embryo-sac structure, and embryo sac fertility was investigated according to Shahid et al. (2010) [9]. Seed setting was counted according to the method of Shahid et al. (2013b) [28].

### Genome re-sequencing

Young Leaves of neo-tetraploid rice lines and autotetraploid rice parents were collected and stored at -80°C for DNA isolation. Genomic DNA was extracted using a modified CTAB method [29]. Sequencing library was prepared according to the standard protocol of Illumina. Then pair-end sequencing was conducted by Illumina HiSeqTM 2500 and Hiseq X Ten platform (BioMarkers, Beijing, China). The generated FASTQ file quality was evaluated using FastQC (http://www.bioinformatics.babraham.ac.uk/projects/fastqc/). Then low quality reads, including reads with sequencing adapter, reads with more than 10% N content, and reads with more than 50% low quality bases (<10), were filtered. After filtration, clean data were aligned to the Nipponbare reference genome (http://plants.ensembl.org/Oryza_sativa/Info/Index) by Burrows-Wheeler Aligner (BWA) software [30]. MarkDuplicates in Picard (https://sourceforge.net/projects/picard/) was used to eliminate the PCR duplication. We used Genome Analysis Toolkit (GATK) for base recalibration and realignment near insertion or deletion regions. SAMtools was used to estimate reference genome coverage [31].

### Identification and analysis of variations

The filtered alignment files were used for the identification of SNPs and InDels. The following SNPs and InDels were filtered: two or more SNPs in a 5 bp or shorter window, SNPs near (5 bp or less) InDel, and two or more InDels in a 10 bp or shorter window. We further retained the SNPs and InDels with a coverage depth ranged from 5 to 100. Presence/absence variations (PAVs) were identified by using the BreakDancer software, and structural variations (SVs) with a coverage depth ranged from 6 to 100 were retained [32]. The SNPEFF software was used to annotate SNPs and InDels, and PAVs were annotated based on the GFF file of Nipponbare reference genome [33].

### RNA-seq analysis

Anthers were collected from autotetraploid rice parents and three neo-tetraploid rice lines at meiosis stages. Floret lengths at meiosis stage in autotetraploid rice (T44 and T45) and neo-tetraploid rice (134, 66 and H3) were 4.8–5.3 mm, 5.5–6.0 mm, 5.4–5.8 mm, 5.3–5.7 mm and 5.3–5.7 mm, respectively. All samples were collected in three biological replicates and stored at -80°C for RNA isolation. The total RNA from each sample was extracted from the anthers, ovaries and leaves according to the manual instruction of the TRlzol Reagent (Life technologies, USA). The samples from anther of each biological replicate were mixed for RNA extraction. The quantity and quality of each RNA sample was assessed by using 1% agarose gel and a Nanodrop 1000 spectrophotometer (Nanodrop, USA). RNA integrity number and concentration were checked using an Agilent 2100 Bioanalyzer (Agilent Technologies, USA). The mRNA was isolated by NEBNext Poly (A) mRNA Magnetic Isolation Module (NEB). The enriched and purified mRNA was broken into approximately 200nt short RNA inserts, which were used to synthesize the first-strand cDNA and the second cDNA. The double-stranded cDNA were used to perform end-repair/dA-tail and adaptor ligation. The suitable fragments were isolated by AgencourtAMPure XP beads (Beckman Coulter, Inc.), and enriched by PCR amplification. Finally, the constructed cDNA libraries of the samples were sequenced on a flow cell using an Illumina HiSeq 2500 sequencing platform.

Transcriptome analysis was done by using reference genome-based reads mapping. Low quality reads, such as adaptor sequences, unknown nucleotides>5%, or Q20 <20% (percentage of sequences with sequencing error rates <1%), were removed by NGSQC software [34]. Then the clean reads were mapped onto the Nipponbare (IRGSP-1.0 pseudomolecule/MSU7) reference genome by using Cufflinks and Cuffmerge software [35,36]. Gene expression levels were estimated using FPKM values (Fragments Per Kilobase of transcript per Million fragments mapped) by the Cufflinks and Cuffmerge software [36,37]. The false discovery rate (FDR) control method was used to identify the threshold of the P-value in multiple tests in order to compute the significance of the differences. Here, only genes with an absolute value of Fold Change≥2 and FDR significance score <0.01 were used for the subsequent analysis.

### Bioinformatic analysis

GO analysis was done by using David analysis tools (http://david.abcc.ncifcrf.gov/home.jsp), the Plant GeneSet Enrichment Analysis Toolkit (http://structuralbiology.cau.edu.cn/PlantGSEA/) and AgriGO tool (http://bioinfo.cau.edu.cn/agriGO/), and significance was expressed as a P-value < 0.05. Venny software was used to identify the overlapped differentially expressed genes in different samples and tissues (http://bioinfogp.cnb.csic.es/tools/venny/).

### Verification analysis

Primer 5 software was used to design the primers, and PCR was performed using PrimeSTAR Max DNA polymerase (TaKaRa) according to the manufacturer’s instructions. Amplification reactions (20μl) contained 30 ng DNA template, 0.15 μmol/L each primer (S1 Table), and 1×PrimeSTAR max Premix. The PCR reaction was programmed as follow: 94°C for 3 min, 35 cycles consisting of 94°C for 15 s, 60°C for 5 s and 72°C for 30 s, and a final extension at 72°C for 5 min. PCR products were examined by agarose gel electrophoresis and sequenced by the Beijing Genomics Institute (Guangzhou China). The sequencing results were assembled by DNAMAN software, and further aligned to the reference genome sequences to validate the variations of polymorphic loci using Bioedit software. Total RNAs obtained from rice anthers were reverse transcribed using the PrimeScript RT reagent kit with gDNA Eraser (TaKaRa). The qRT-PCR was performed using the SYBR Premix Ex Taq II kit (TaKaRa) according to the manufacturer’s instructions. Amplification reactions (20 μl) contained 1 μl of cDNA sample, 10 μl of SYBR Premix Ex Taq (2×), and 0.2 μM of each primer (S2 Table). Actin was used as a reference gene. The PCR cycling conditions comprised one denaturation cycle at 94°C for 90 s, followed by 40 amplification cycles (94°C for 10 s, 61°C for 15 s, and 72°C for 20 s). All qRT-PCR amplifications were carried out in triplicate, and the results are presented as mean ± standard deviations. The relative expression levels of genes were calculated by the 2^–ΔΔCT^ method [38].

## Results

### Morphological and cytological observations of neo-tetraploid rice

**Fig 1:**
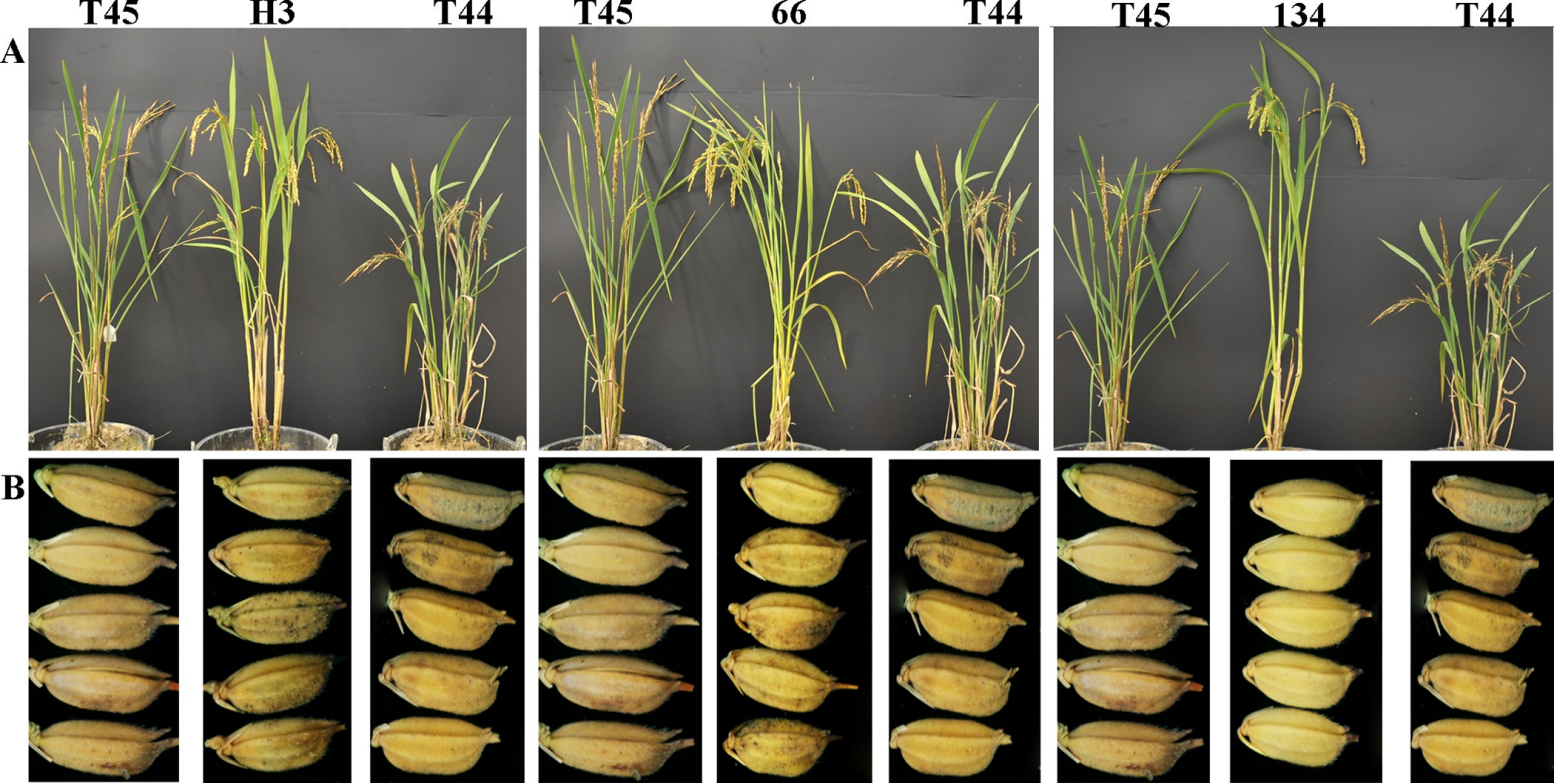
Morphological characteristics of neo-tetraploid rice lines and their parents. (A) Plant appearance of neo-tetraploid rice lines and their parents. Left: Plant structure of 96025 (T44), Huaduo 3 (H3) and Jackson-4x (T45), and H3 was developed from T44 and T45. Middle: Plant structure of 96025 (T44), Huaduo 5 (66) and Jackson-4x (T45), and 66 was developed from T44 and T45. Right: Plant structure of 96025 (T44), Huaduo 4 (134) and Jackson-4x (T45), and 134 was developed from T44 and T45. (B): Grains of neo-tetraploid rice lines and their parents.

**Fig 2:**
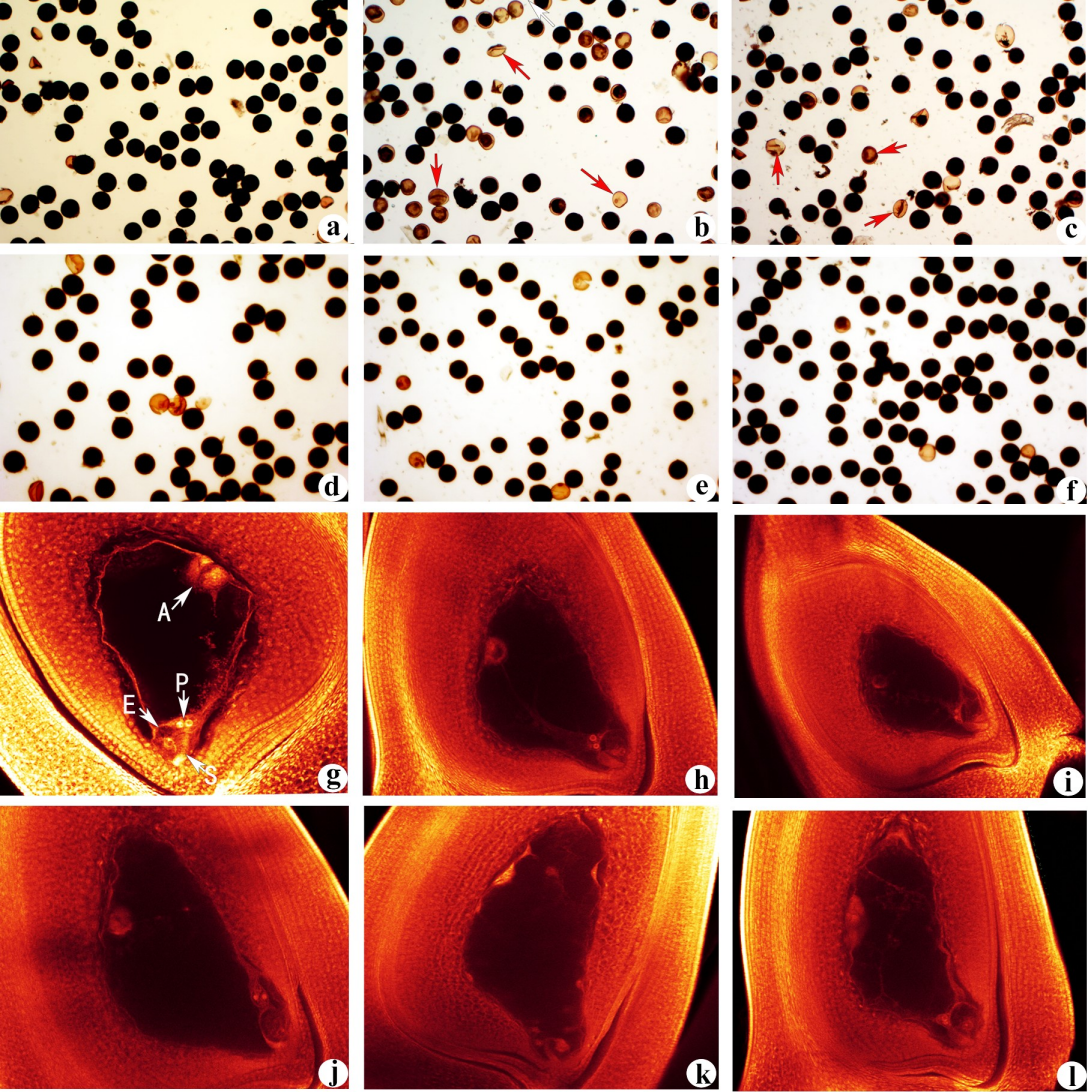
Pollen and embryo sac development in neo-tetraploid rice lines. (a): Pollens of diploid rice (>95% pollen fertility). (b) and (c): Pollens of autotetraploid parents (T44 and T45), Arrows indicated abnormal pollens. (d), (e) and (f): Pollens of neo-tetraploid rice lines, and more than 92% pollens were normal. (g) The normal structure of mature embryo sac in diploid rice, showing the egg cell (E), synergid (S), polar nucleus (P) and antipodal (A). (h) and (i) The normal structure of mature embryo sac in autotetraploid parents. (j), (k) and (l): The normal structure of mature embryo sac in neo-tetraploid rice lines.

A total of 20 plants from each autotetraploid rice parent and three neo-tetraploid rice lines were harvested from the field at maturity. Three neo-tetraploid rice lines displayed significant differences in agronomic traits, including plant height, number of filled grains per plants, 1000-grain weight and seed setting (Fig 1, Table 1). Importantly, the seed setting and pollen fertility of neo-tetraploid rice lines were significantly higher than their parents. Three neo-tetraploid rice lines produced 68.3%, 68.1% and 71.8% seed set, while it was only 31.5% and 25.4% in autotetraploid parents (Table 1). Pollen fertility of neo-tetraploid rice lines was more than 92%, while parents produced 65.83% and 76.75% pollen fertility (Fig 2, Table 1). Non-significant differences were detected in embryo sac fertility between neo-tetraploid rice lines and autotetraploid parents (Table 1), and normal embryo sacs of parents and neo-tetraploid lines are shown in the Fig 2. Main embryo sac abnormalities were the number and location of abnormal polar nuclei, degenerated embryo sac, the degradation of female reproductive units and egg apparatus degradation in autotetraploid parents and three neo-tetraploid rice lines. The seed setting of hybrid lines generated by crossing with various autotetraploid lines were significantly higher than autotetraploid parents (S3 Table).

**Table 1:** Agronomic traits of neo-tetraploid rice lines and their autotetraploid parents.

Traits/Names	T44	T45	H3	66	134
Plant height (cm)	82.7±6.03	99±5.31	101.8±2.1	114±10.8	151.8±11.3
Number of panicles	3.2±0.84	3.4±0.55	3.8±0.8	4±0.71	2.8±0.84
Panicle length (cm)	31.4±1.95	33±3.2	25.5±1.2	23.8±2.16	29.5±1.87
Number of Grains per plant	383.4±42.8	385.6±78.8	392.6±47.5	330.2±49.3	413.6±69.2
Number of filled Grains per plant	120.2±47.8	96.2±20.4	268.2±32.1	222.4±30.2	295.6±49.1
1000- grain weight (g)	25.1±0.97	25.3±1.54	31.5±1.9	29.2±2.3	33.2±1.3
Seed setting (%)	31.5±12.3	25.4±5.1	68.3±2.6	68.1±2.1	71.8±6.5
Pollen fertility (%)	65.80	76.75	94.29	92.29	93.86
Embryo sac fertility (%)	89.44	88.62	88.78	90.36	90.61

Note: 134, 66 and H3 represent neo-tetraploid rice lines, including Huaduo 4, Huaduo 5 and Huaduo 3, respectively; T44 and T45 represent autotetraploid rice parents, including Jackson-4x and 96025, respectively.

**Fig 3:**
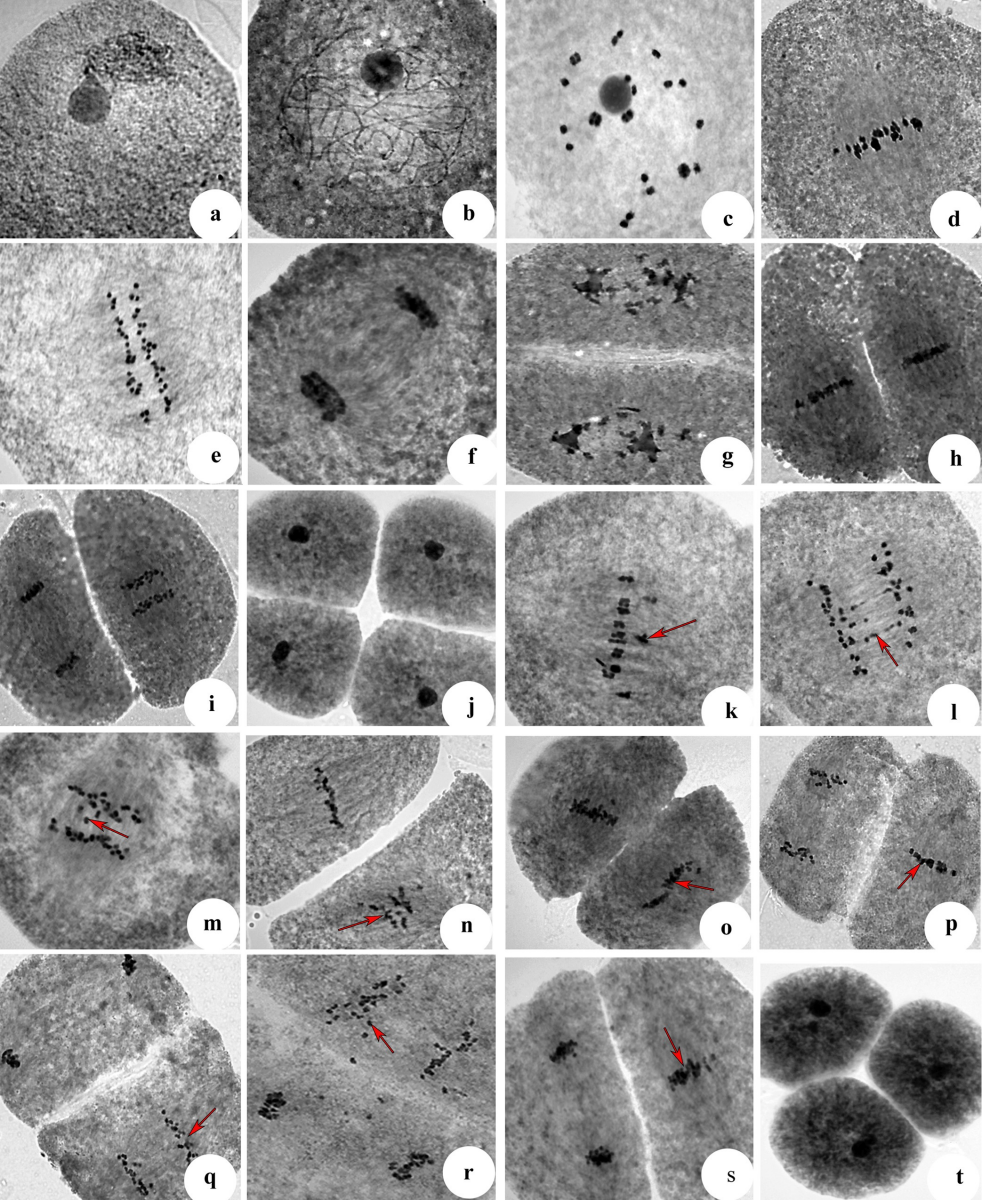
Chromosome behavior during PMC meiosis in neo-tetraploid rice. (a): Leptotene, (b) Pachytene, (c) Diakinesis, (d) Metaphase I, (e) Anaphase I, (f) Telophase I, (g) Prophase II, (h) Metaphase II, (i) Anaphase II, (j) Tetrad. (k) Chromosome lagging at metaphase (arrow), (l) Anaphase I chromosome bridge (arrow), (m) Anaphase I chromosome straggling (arrow), (n) Metaphase II chromosome lagging (arrow), (o) Metaphase II distorted spindle (arrow), (p) Abnormal anaphase II showing asynchronous cell division (arrow), (q) Anaphase II showing asynchronous cell division (arrow), (r) and (s) Abnormal telophase II showing asynchronous cell division (arrow), (t) Triad.

Chromosome behavior observation revealed that the meiotic stages of neo-tetraploid rice lines were similar to their autotetraploid parents and diploid rice, which could be divided into nine stages, including prophase I (leptotene, zygotene, pachytene, diplotene and diakinesis (Fig 3A–3C), metaphase I (Fig 3D), anaphase I (Fig 3E), telophase I or dyad (Fig 3F), prophase II (Fig 3G), metaphase II (Fig 3H), anaphase II (Fig 3I), telophase II and tetrad (Fig 3J). Many abnormalities, including multivalent, chromosome straggling and lagging, distorted spindles, asynchronous cell division and triad, were observed in autotetraploid parents (Table 2, Fig 3K–3T). However, few abnormalities were detected in neo-tetraploid rice compared to their parents, which displayed different chromosomal configurations at diakinesis (Table 2, Table 3). These results indicated an association between chromosome behavior and high fertility in neo-tetraploid rice.

**Table 2:** Frequency of abnormal chromosome behaviors during meiosis in neo-tetraploid rice lines and their autotetraploid parents.

Stages		T45		T44		66		H3		134	
		No. of cells	Abnormal cell (%)	No. of cells	Abnormal cell (%)	No. of cells	Abnormal cell (%)	No. of cells	Abnormal cell (%)	No. of cells	Abnormal cell (%)
Meiosis I	Metaphase I	295	55.9	208	52.1	350	17.1	278	20.1	212	17.9
	Anaphase I	150	21.3	186	15.1	277	18.7	264	15.9	202	15.8
	Telophase I	216	16.7	228	29.3	274	10.2	266	9.7	216	3.2
Meiosis II	Metaphase II	191	25.1	303	23.1	224	25.4	300	33.0	207	31.4
	Anaphase II	171	58.9	153	37.9	209	21.1	304	29.6	154	22.1
	Telophase II	156	12.2	231	12.1	236	12.3	282	14.8	206	9.2
	Tetrad	153	1.3	271	5.2	192	0	213	0	218	0

Note: 134, 66 and H3 represent neo-tetraploid rice lines, including Huaduo 4, Huaduo 5 and Huaduo 3, respectively; T44 and T45 represent autotetraploid rice parents, including Jackson-4x and 96025, respectively.

**Table 3:** Meiotic chromosome configurations in neo-tetraploid rice lines and their autotetraploid parents.

	No. of cells	Chromosome configuration
T45	123	(0.55±0.10) I + (7.93±0.27) II + (0.07±0.02) III+ (7.16±0.14) IV +(0.03±0.01) V +(0.27±0.05) VI +(0.12±0.03)VIII
T44	112	(0.34±0.09) I + (5.55±0.40) II+ (0.04±0.02) III+ (8.68±0.19) IV+(0.22±0.05) VI+(0.05±0.03) VIII
H3	102	(0.53±0.09) I + (9.79±0.46) II+ (0.16±0.04) III+ (6.85±0.23) IV
66	109	(0.40±0.08) I + (9.27±0.34) II+ (0.11±0.03) III+ (7.18±0.17) IV
134	104	(0.11±0.03) I+ (9.73±0.28) II + (0.37±0.03) III+ (7.01±0.13) IV

Note: 134, 66 and H3 represent neo-tetraploid rice lines, including Huaduo 4, Huaduo 5 and Huaduo 3, respectively; T44 and T45 represent autotetraploid rice parents, including Jackson-4x and 96025, respectively; I, II, III and IV represent univalent, bivalent, trivalent and tetravalent, respectively.

### Discovery of SNPs and InDels in neo-tetraploid rice

A total of 447 million reads were generated for neo-tetraploid rice lines and its autotetraploid parents. After the removal of low-quality reads, about 98% of the reads were retained as clean data and used for further investigation. The high quality reads were further mapped onto the reference genome (MSU7.0) using BWA software. Overall, almost 92% of these reads were uniquely mapped, and covered about 90% of the reference genome with at least 10× coverage depth, and the average coverage depth was approximately 90% in three neo-tetraploid rice lines and its autotetraploid parents (S4 Table).

Compared with the reference genome sequence, a total of 4750473 polymorphic sites including 4109881 SNPs and 640592 InDels were discovered in neo-tetraploid rice lines. We applied following two criteria to decrease the rate of false-positive SNPs and InDels: 5< read depths > 100; sequencing score of 30 (Q30), which indicates an error rate of one per 1000 reads. Based on these filter conditions, the total number of DNA polymorphisms were 1104015 to 1631038 in three neo-tetraploid rice lines (134, 66 and H3) and two parents, and the percentages of heterozygous DNA polymorphisms were 13.4%, 10.8%, 16.7%, 11.8% and 6.8%, respectively (S5 Table).

Further, we identified SNPs and InDels between three neo-tetraploid rice lines and their autotetraploid parents. The numbers of DNA polymorphisms were about 1.38, 1.36 and 1.19 times higher for 134 vs T44, 66 vs T44 and H3 vs T44 as compared to 134 vs T45, 66 vs T45 and H3 vs T45, respectively. The total numbers of SNPs were 133187, 107978 and 167136 for neo-tetraploid lines (134, 66 and H3) compared to two parents and the percentages of heterozygous SNPs were 73.54%, 51.96% and 79.11%, respectively. In total, 79259, 8085 and 26236 InDels were detected in neo-tetraploid lines (134, 66 and H3) compared to two parents, and the percentages of heterozygous SNPs were 9.6%, 52.1% and 38.52%, respectively (S6 Table). The sequencing results were further validated by PCR amplification, and 76 randomly-selected variations sites were sequenced. The results showed that the DNA variation sites by Sanger sequencing were consistent with the re-sequencing data (S7 Table).

### Genomic distribution and analysis of SNPs and InDels in neo-tetraploid rice

**Fig 4:**
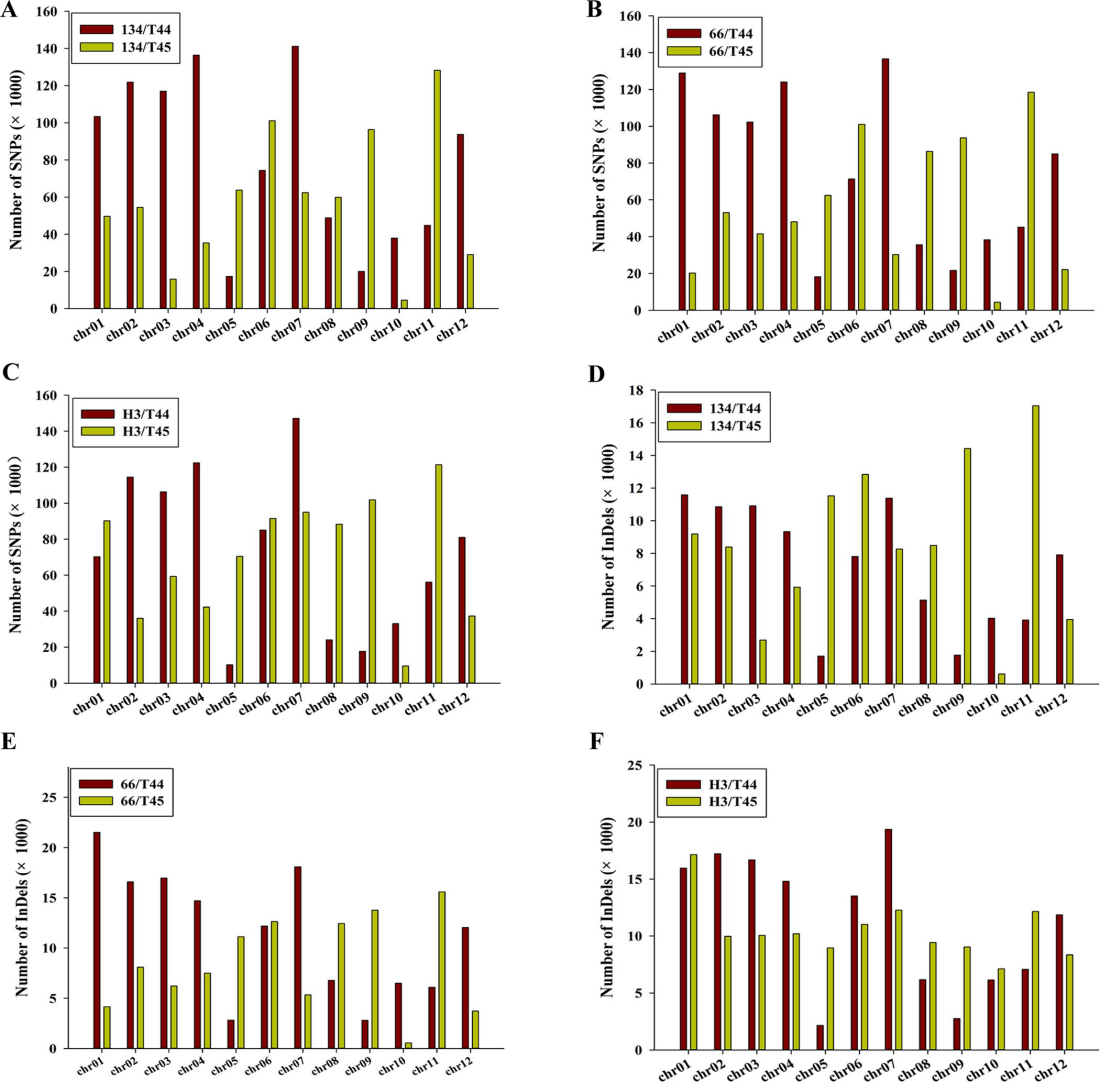
Number and distribution of single-nucleotide polymorphisms (SNPs) and insertions/deletions (InDels) detected on the rice chromosomes. Total number of SNPs (A, C, E) and InDels (B, D, F) detected on each rice chromosome are shown in the bar graphs.

The distribution of DNA polymorphisms in neo-tetraploid rice/autotetraploid parents was analyzed across the twelve rice chromosomes, and the results indicated that total number of SNPs and InDels on a chromosome were proportional to chromosome length (S8 Table and S9 Table). In neo-tetraploid rice vs T44, the largest number of SNPs was detected on chr7, while chr5 had the smallest number of SNPs. Similarly, the higher numbers of InDels were observed on chr7, chr1 and chr7, while chr5, chr9 and chr9 had smaller numbers of InDels in 134, 66 and H3, respectively. The highest SNP density was found on chr7. In neo-tetraploid rice lines relative to T45, the largest numbers of SNPs were detected on chr11, while chr10 had the smallest number of SNPs. Similarly, the highest numbers of InDels were observed on chr11, chr11 and chr1 in 134, 66 and H3, while chr10 had the smallest number of InDels in three neo-tetraploid rice compared to T45. The highest SNP frequency was detected on chr7 chr9 and chr10 in 134, 66 and H3 compared to T45, respectively (Fig 4).

Moreover, we observed that SNPs were not uniformly distributed across the chromosomes. In neo-tetraploid rice lines (134, 66 and H3) compared to T44, a total of 1302, 905 and 1162 high-density (>250) SNP regions of 100kb were identified. Similarly, a total of 243, 476 and 320 low-density (<5) SNP regions of 100 kb were detected in H3, 66 and 134, respectively. In neo-tetraploid rice lines compared to T45, 931, 1529 and 1138 high-density (>250) SNP regions, and 462, 78 and 362 low-density (<5) SNP regions of 100 kb were detected. The genomic regions with no SNP were also detected in neo-tetraploid rice lines.

The frequency of transition (A/G and C/T; Ts) was much higher than transversions (A/C, A/T, G/C, and G/T; Tv), and the ratio of Ti/Tv was 2.58, 2.60, 2.58 in neo-tetraploid rice lines (134, 66 and H3) compared to T44, while it was 2.62, 2.64 2.65 in neo-tetraploid rice lines compared to T45 (Fig A in S1 File). The frequency of both A/G and C/T transitions was similar. However, among transversions, the frequency of A/T was higher than G/C. Further, analysis on the length distribution of InDels detected in neo-tetraploid rice/autotetraploid parents displayed that about half of InDels (48.9%) were 1 bp (mononucleotide insertion-deletion), 32% were 2–5 bp and 20% were ≥6 bp (Fig A in S1 File).

PAVs (>100bp) are a major source of genome structural variation and have profound effects on phenotypic and genomic variation in plants. So, we further analyzed PAVs in neo-tetraploid rice. A total of 596, 644 and 565 specific PAVs were detected in neo-tetraploid rice lines compared to autotetraploid parents, and these PAVs influenced the length of chromosome in varying degrees. The highest numbers of PAVs were detected on chr1, chr1, and chr7, while the smaller chromosomes, such as chr12, chr10 and chr10, had the lowest number of PVAs in H3, 66 and 134, respectively. However, the biggest influence of chromosome size was detected on chr9 as well as on the smallest chr10, chr3 and chr4 of H3, 66 and 134, respectively (S10 Table).

### Annotation and effect of SNPs and InDels on amino acid substitution in neo-tetraploid rice

The annotation of rice genome revealed the distribution of SNPs and InDels within various genomic regions, such as intergenic and intragenic. Overall, a similar distribution pattern of SNPs and InDels was observed in neo-tetraploid rice/ autotetraploid parents (Fig B-D in S1 File). Approximately 50% of SNPs were identified in intergenic region. About 12% of the total SNPs were detected in the genic regions, and significant proportions of SNPs were detected in 2 kb upstream and 1 kb downstream regions. Within the genic region, more than 6% of SNPs were present in the introns. The 3’UTR and 5’UTR regions also showed the presence of SNPs (0.5–1.0%). Similarly, about 42% of InDels were identified in intergenic regions in both types of rice. Only 0.5% of InDels were present in the exonic regions, whereas upstream and downstream regions contained about 20% InDels. Within the genic region, almost 7% of InDels were present in the introns. Similar to SNPs, InDels (0.3–0.8%) were also observed in 3’UTR and 5’UTR regions.

The reasons for high seed setting of three neo-tetraploid rice lines were common mutated sites, so we further analyzed the mutated genes of same site that might be associated with fertility in three neo-tetraploid rice lines. A total of 9397 and 6980 genes, harboring at least one of the SNPs or/and InDels, exhibited mutations in neo-tetraploid rice lines compared to T44 and T45, respectively, and 1362 genes showed variations in neo-tetraploid rice lines compared to their parents, and variation sites were present in upstream, downstream, intron, and coding regions.

We analyzed the effect of SNPs on amino acid substitution, and high proportion of the SNPs in CDS region was found to be non-synonymous in neo-tetraploid rice lines and autotetraploid parents. These non-synonymous substitutions were present in 3622 genes in neo-tetraploid rice lines compared to T44 and 2804 genes compared to T45. Of the mutated genes, 181 genes were present in both groups i.e. neo-tetraploid rice lines vs T44 and neo-tetraploid rice lines vs T45 (S11 Table).

We further analyzed the distribution of large-effect SNPs and InDels, which are predicted to have a pronounced effect on the loss of gene function. A total of 305 and 224 large-effect SNPs loci in neo-tetraploid rice lines were detected compared to T44 and T45, respectively. Of these, 291 and 208 genes affected the integrity of encoded proteins in neo-tetraploid rice lines (Table 4). The large-effect SNPs included disruption of splice sites, loss of translation initiation codon, introduction of premature stop codon and loss of stop codon. Similarly, we identified 441 and 392 InDels in 315 and 269 genes, which cause frame shift, disruption of splice sites or introduction of premature stop codon (Table 4). Overall, 545 and 441 genes harbored at least one large-effect SNP and/or InDel. Among these genes, 28 common mutated genes were found in neo-tetraploid rice lines compared to T44 and T45 (S12 Table). These genes were not found to be involved in any biological process.

**Table 4:** Large-effect SNPs and InDels detected in neo-tetraploid rice and its autotetraploid parents.

	Neo-tetraploid rice/T44		Neo-tetraploid rice/T45	
	SNP	INDEL	SNP	INDEL
Acceptor splice site	22	8	17	13
Donor splice site	23	9	19	13
Start lost	29	4	26	5
Stop gained	169	2	116	6
Stop lost	62	2	46	4
Frame shift	0	416	0	351
Total	305	441	224	392

Note: T44 and T45 represent autotetraploid rice parents, including Jackson-4x and 96025, respectively.

### New mutation in neo-tetraploid rice

New mutations in neo-tetraploid rice are different compared to their parents at the same site and might be associated with fertility in neo-tetraploid rice. So, we further investigated the existence of new mutations in neo-tetraploid rice lines, and many new SNPs and InDels were detected in neo-tetraploid rice genome. Of 1362 mutated genes, 324 genes harbored at least one new peculiar variation site in neo-tetraploid rice lines (S13 Table), and we focused on these 324 important genes. GO analysis revealed that these 324 genes were significantly enriched in polar nucleus fusion, RNA 3'-end processing, cellular protein modification process, phosphorylation, and protein ubiquitination (S14 Table). Co-expression analysis revealed that 19 of the 324 specific mutated genes were co-expressed genes. Of these 19 putatively co-expressed genes, the biological functions of Os05g0209000, Os06g0558900, Os11g0513700 and Os11g0513900 are still unknown. The other genes were Os01g0715600 (auxin efflux carrier component), Os05g0208550 (gibberellin 2-beta-dioxygenase 1), Os05g0212200 (Leucine Rich Repeat family protein), Os05g0211100 (cytochrome P450), Os05g0519700 (heat shock protein), Os06g0549900 (reticuline oxidase-like protein precursor), Os06g0552900 (FT-Like12 homologous to Flowering Locus T gen), Os06g0553800 (plastocyanin-like domain containing protein), Os06g0603600 (SPX domain containing protein), Os06g0604000(AP2 domain containing protein), Os11g0514500 (brassinosteroid insensitive 1-associated receptor kinase 1 precursor), Os11g0562100 (cycloartenol synthase), Os11g0565300 (OsWAK receptor-like protein kinase), Os12g0553200 (RGH1A), and Os12g0559200 (lipoxygenase 2.1).

Meiosis is a vital process during pollen development and low pollen fertility and abnormal chromosome behaviors were observed in autotetraploid rice. We focused on the polymorphic genes that could be associated with meiosis by comparing with meiosis-related and stage-specific genes reported in rice and other plants [18,38,39]. Of these 324 genes, we found 52 meiosis-related genes (S15 Table), but their functions are unknown during meiosis. Moreover, we detected eight epigenetics related genes (Table 5). Of these genes, one codon insertion and one synonymous variation were detected in Os06g0535200. One intron variation and codon deletion were detected in Os06g0537500. Two non-synonymous SNP variations and one frame shift mutation were identified in Os01g0719100, and all aforementioned genes annotated E3 ubiquitin-protein ligase. Os05g0392400 annotated SNF2 domain-containing protein, which had three mutations in intron region. One non-synonymous SNP was identified in Os08g0289400, which annotated Serine/arginine-rich splicing factor SR45. Two synonymous SNP variations were detected in Os10g0357800, which annotated N-dimethylguanosine tRNA methyltransferase, and one synonymous SNP variation was detected in Os12g0211400 that annotated adenine DNA glycosylase. Two non-synonymous SNP variations and one frame shift mutation were detected in Os04g0572600, which encoded DNA-directed RNA polymerase IV subunit 1. The tissue-specific analysis indicated that Os06g0535200 was not expressed in anther, and specifically expressed in root, while Os05g0392400 was specifically expressed in anther. The highest amount of Os01g0719100 transcripts were detected in embryo and anther, and Os04g0572600, Os10g0357800 and Os12g0211400 displayed high levels of expressions in anther, panicle and inflorescence, respectively.

**Table 5:** Important mutant genes in neo-tetraploid rice.

ID	Gene name	Mutation site	Expression in neo-tetraploid rice
*Os06g0535200*	E3 ubiquitin-protein ligase	CDS	No differential expression
*Os01g0719100*	E3 ubiquitin-protein ligase	CDS	Differentially expressed
*Os06g0537500*	E3 ubiquitin-protein ligase	intron and CDS	No differential expression
*Os08g0289400*	Serine/arginine-rich splicing factor SR45	CDS	No differential expression
*Os04g0572600*	DNA-directed RNA polymerase IV subunit 1	CDS	Differentially expressed
*Os10g0357800*	N-dimethylguanosine tRNA methyltransferase	CDS	No differential expression
*Os12g0211400*	adenine DNA glycosylase	CDS	No differential expression
*Os05g0392400*	SNF2 domain-containing protein	intron	Differentially expressed

**Fig 5:**
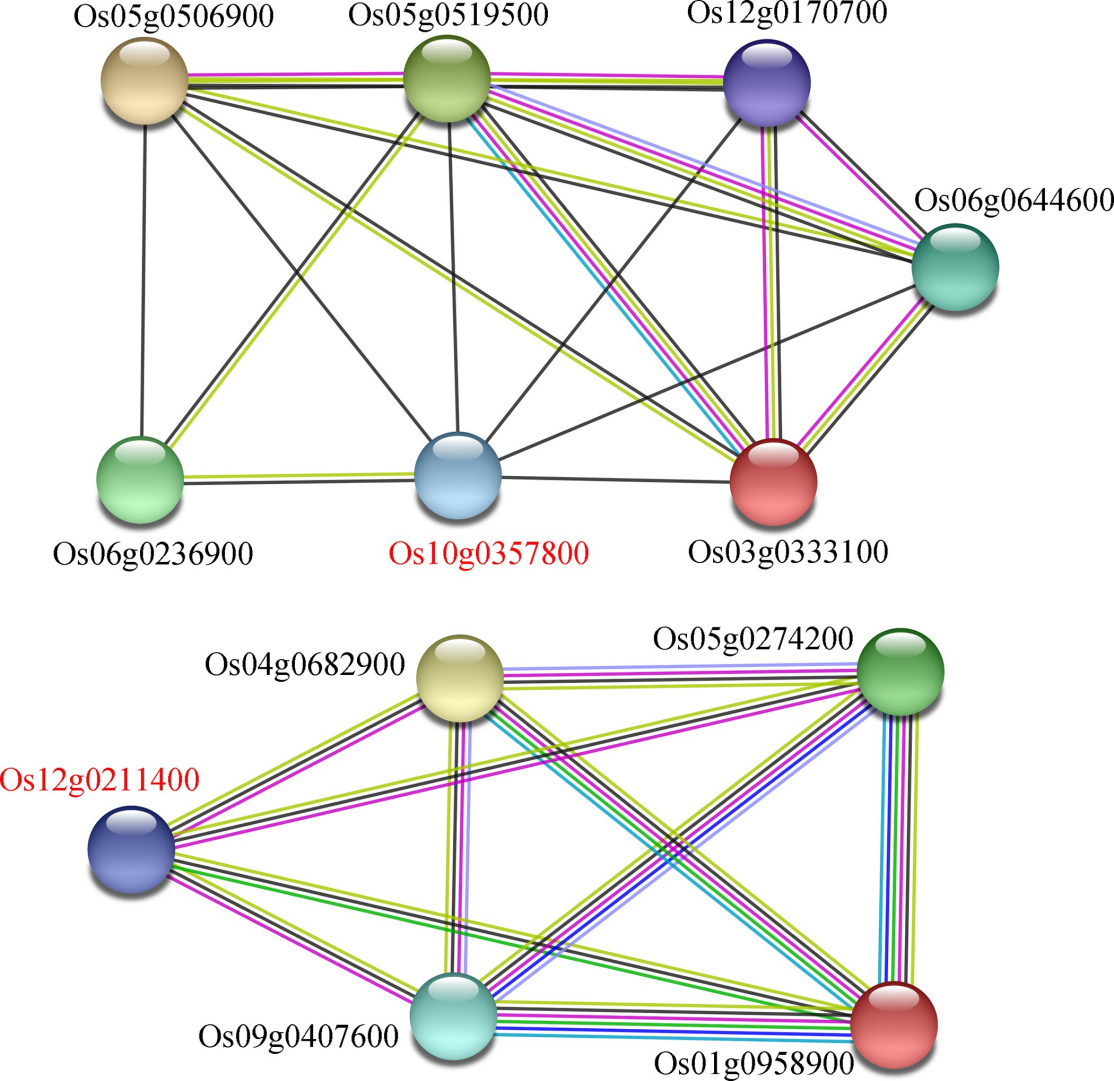
Predicted protein-protein interaction network of mutated and fertility-related genes. Predicted protein-protein interaction subnetwork was constructed using mutated genes between autotetraploid and neo-tetraploid rice lines and fertility-related genes reported in rice and other plants. Red and Black letters indicate mutated and fertility related genes, respectively.

We performed the protein-protein interactions of cloned fertility related genes and specific mutated genes using STRING v10. We identified two mutated genes that were associated with the meiosis related genes (Fig 5). Among the mutated genes, Os10g0357800 showed interactions with Os04g0112300 (tRNA methyltransferase), Os12g0170700 (N-acetyltransferase 10), Os05g0519500 (G-beta repeat domain containing protein), Os03g0333100 (AARP2CN domain containing protein), Os03g0699200 (BRCA1 C Terminus domain containing protein), Os06g0236900 (tRNA methyltransferase), Os06g0644600 (WD repeat-containing protein), Os06g0498500 (NOC3—Putative nucleolar complex subunit 3) and Os05g0506900 (Brix domain-containing protein 1). Os12g0211400 showed interactions with Os09g0407600 (MSH-like DNA mismatch repair protein), Os05g0274200 (MSH-like DNA mismatch repair protein), Os04g0682900 (DNA mismatch repair protein MSH3) and Os01g0958900 (DNA mismatch repair protein Mlh1). But the expression patterns of these two peculiar mutated genes displayed non-significant changes between neo-tetraploid rice and autotetraploid rice by qRT-PCR.

### The changes in gene expression patterns detected by RNA-seq during meiosis

To further investigate the influence of mutations and transposon elements on the expression of genes, transcriptome sequencing was used to detect the putative and meiosis related genes during meiosis. Genes showed more than two fold up- or down-regulation between the neo-tetraploid rice lines and parents were classified as ‘‘differentially expressed genes (DEGs)”. In total, 3471, 3117 and 3794 genes showed differential expression patterns between three neo-tetraploid rice lines and T44. Of these genes, 1905, 1969, 2030 and 1566, 1148, 1764 were found to be up- and down-regulated in 134, 66 and H3, respectively. In neo-tetraploid rice lines compared to T45, 2371, 1929 and 4448 genes showed differential expressions in 134, 66 and H3 respectively. Of these genes, 924, 746, 2376 and 1447, 1183, 2072 were up- and down-regulated, respectively. The reason for high seed setting of all neo-tetraploid rice lines is that they may have common differentially expressed genes during meiosis. So, we further analyzed common differentially expressed genes in three neo-tetraploid rice lines by Venn analysis. 1473 and 766 genes were common and differentially expressed in neo-tetraploid rice lines compared to T44 and T45 (S16 Table, S17 Table).

Of 1473 DEGs, 132 genes were noncoding RNAs (ncRNA) and 41 genes were transposon elements (S18 Table, S19 Table). 129 genes only expressed in neo-tetraploid rice, while 177 genes only expressed in T44 (S20 Table). mRNA level of 23 specific mutant genes exhibited significant changes in neo-tetraploid rice (S21 Table). Among these 23 genes, 8 meiosis related genes were differentially expressed, including Os01g0716200 (uncharacterized in meiosis stage), Os01g0719100 (E3 ubiquitin-protein ligase, but uncharacterized in meiosis stage), Os05g0519300 (uncharacterized in meiosis stage), Os05g0527700 (uncharacterized in meiosis stage), Os06g0556300 (uncharacterized in meiosis stage), Os06g0559400 (uncharacterized in meiosis stage), Os11g0513900 (uncharacterized in meiosis stage) and Os11g0558400 (uncharacterized in meiosis stage). Meanwhile, three specific mutant epigenetics related genes also differentially expressed in neo-tetraploid rice lines compared to both parents, and Os01g0719100 was found to be down-regulated in neo-tetraploid rice lines, while Os04g0572600 and Os05g0392400 were up-regulated in neo-tetraploid rice lines.

Of 766 DEGs that were differentially expressed in neo-tetraploid rice lines compared to T45, 12 genes were related to transposon elements and 113 genes were identified as ncRNA (S22 Table, S23 Table). 22 genes only expressed in neo-tetraploid rice, while 173 genes only expressed in autotetraploid rice (S24 Table). mRNA level of 12 specific mutant genes depicted significant changes in neo-tetraploid rice (S25 Table). Among these 12 genes, eight meiosis related genes were differentially expressed, including Os01g0716200 (uncharacterized in meiosis stage), Os01g0716300 (uncharacterized in meiosis stage), Os01g0719100 (E3 ubiquitin-protein ligase, uncharacterized in meiosis stage), Os05g0210700 (uncharacterized in meiosis stage), Os05g0527700 (uncharacterized in meiosis stage), Os06g0556300 (uncharacterized in meiosis stage), Os06g0601100 (uncharacterized in meiosis stage) and Os11g0513900 (uncharacterized in meiosis stage).

Moreover, 57 genes were differentially expressed in neo-tetraploid rice lines compared to their parents, including 7 specific mutant genes. Of these 7 genes, 5 meiosis related genes were differentially expressed, including Os01g0716200, Os01g0719100, Os05g0527700, Os06g0556300 and Os11g0513900, Epigenetics related genes, including Os04g0572600 (DNA-directed RNA polymerase IV subunit 1) and Os05g0392400 (SNF2 domain-containing protein) were up-regulated, while Os01g0719100 (E3 ubiquitin-protein ligase) was down-regulated (S26 Table). These results suggested that specific mutations affect genes expression and function, which might be associated with fertility in neo-tetraploid rice.

**Fig 6:**
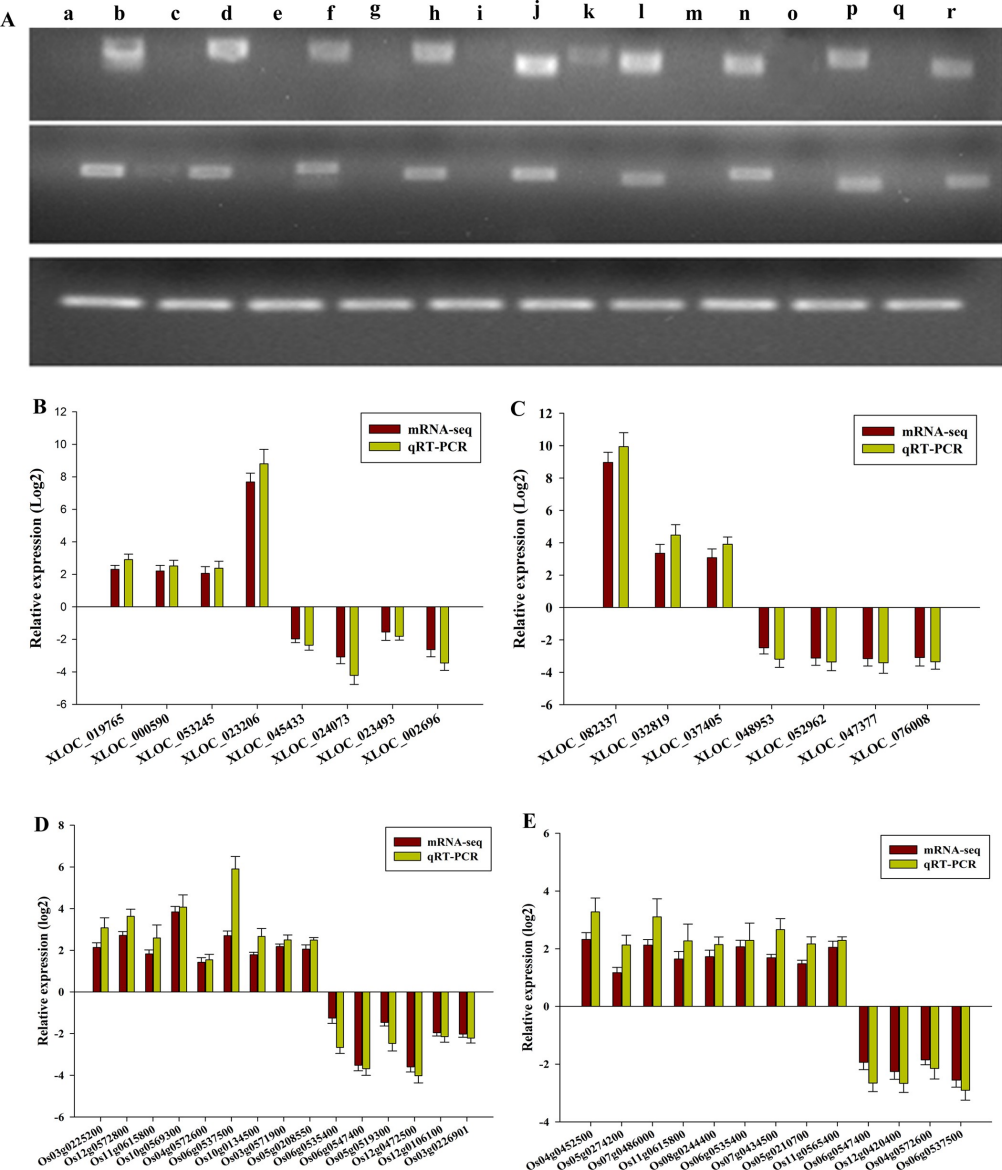
Confirmation of mRNA-seq data by qRT-PCR in neo-tetraploid rice and autotetraploid rice during PMC meiosis. (A) The specific expression patterns of genes in neo-tetraploid rice or autotetraploid rice. Upper lane: specific expressed genes in neo-tetraploid rice, a, c, e, g, i, k, m, o and q indicated the genes only expressed in autotetraploid parents, b, d, f, h, j. l, n, p, and r indicated the genes only expressed in neo-tetraploid rice lines. Middle lane: specific expressed genes in neo-tetraploid rice, a, c, e, g, i, k, m, o and q indicated the genes only expressed in neo-tetraploid rice lines, b, d, f, h, j. l, n, p, and r indicated the genes only expressed in autotetraploid parents. The lower lane indicated the expression of reference gene in neo-tetraploid rice lines and autotetraploid parents. (B) and (C) represent differentially expressed ncRNA in neo-tetraploid rice compared to autotetraploid rice, (B): the differentially expressed ncRNA in neo-tetraploid rice lines compared to T44, (C): the differentially expressed ncRNA in neo-tetraploid rice lines compared to T45. (D) and (E) represent the differentially expressed genes in neo-tetraploid rice lines compared to autotetraploid rice. (D): the differentially expressed genes in neo-tetraploid rice lines compared to T44, (E): the differentially expressed genes in neo-tetraploid rice lines compared to T45.

To confirm the expression levels of differentially expressed genes in neo-tetraploid rice and autotetraploid rice, 61 genes were selected for qRT-PCR analysis at meiosis stage, including nine genes only expressed in autotetraploid rice, nine genes only expressed in neo-tetraploid rice, 18 up-regulated genes in neo-tetraploid rice, 10 down-regulated genes in neo-tetraploid rice, seven up-regulated ncRNA in neo-tetraploid rice, and eight down-regulated ncRNA in autotetraploid rice. Based on the qRT-PCR analysis, the expression patterns of all these genes were consistent with RNA-seq data (Fig 6).

## Discussion

### Mutations in novel meiosis and epigenetic-related genes may associate with fertility in neo-tetraploid rice

Meiosis plays significant roles in the life cycle of all sexually propagating eukaryotes, and a number of key genes have been identified and functionally studied in rice and other plants [39, 40]. Here, four genes showed specific mutations and differentially expressed between neo-tetraploid rice and autotetraploid rice during meiosis, but their functions are unknown, including Os01g0716200, Os05g0527700, Os06g0556300 and Os11g0513900. Moreover, these genes were also found to be differentially expressed during meiosis in the previous studies [13,18,19]. It indicates that Os01g0716200, Os05g0527700, Os06g0556300 and Os11g0513900 might be related to fertility in neo—tetraploid rice. Protein ubiquitination is post-translations modification, and it has been demonstrated that components of the ubiquitin system are involved in the regulation of a specific protein’s degradation [41,42]. E3 ubiquitin-protein ligase, a multi-protein complex, is responsible for targeting ubiquitination to specific substrate proteins [43]. Ubiquitination has been demonstrated to be involved in chromosome segregation and polar body extrusion [44,45,46]. Os01g0719100, annotated E3 ubiquitin-protein ligase, displayed specific mutation and differentially expressed between neo-tetraploid rice and autotetraploid rice, which indicated that Os01g0719100 may play a key role during meiosis in neo-tetraploid rice. These mutations altered the expression level of Os01g0719100 and even functions, which affected modifications of meiosis related protein.

SNF2 domain-containing protein, RDR2 and NRPD1a are required for the production of endogenous 24-nucleotide short interfering RNAs in *Arabidopsis thaliana* [47]. The 24nt-siRNA regulates epigenetic silencing by directing DNA methylation through RNA-directed DNA methylation pathway [5,48,49]. Recent studies revealed that 24nt-siRNA related to DNA methylation of class II transposable elements suppressed the expression of nearby genes in autotetraploid rice that were involved in pollen and embryo sac fertility [2,5]. The highest amount of Os05g0392400 (SNF2 domain-containing protein) transcripts were detected in anther, which suggests that Os05g0392400 play an important role in anther development. Three specific mutations were found in intron, and the expression of Os05g0392400 was up-regulated in three neo-tetraploid rice lines. This result suggests that three intron variations may affect the level of Os05g0392400 methylation and changed expression levels, which affected the expression levels of some fertility-related 24nt-siRNA in neo-tetraploid rice.

The components of RNA polymerase IV mediate short-interfering RNAs (siRNAs) accumulation and subsequent RNA-directed DNA methylation-dependent transcriptional gene silencing of target sequences [50,51,52]. Some studies suggested that siRNAs are essential to regulate the genes expression and play a crucial role in male meiosis and pollen development [2,5]. Os04g0572600, annotated DNA-directed RNA polymerase IV subunit 1, had two non-synonymous SNP variations and one frame shift mutation, and the mRNA level of Os04g0572600 markedly change in neo-tetraploid rice. We speculated that these mutations altered the expression pattern of Os04g0572600 and even function, which lead to changes in fertility-related 24nt-siRNA in neo-tetraploid rice.

### Genomic structural reprogramming may associates with fertility in neo-tetraploid rice

Genetic diploidization following whole-genome duplications in plant may have occurred quite frequently during organismal evolution [53]. Earlier reports suggested that chromosomal rearrangements through processes such as neo-functionalization, sub-functionalization or loss of duplicated segments, recombination, transposable elements and genetic drift, cause differences between formerly homologous chromosomes [54,55]. In the present study, more than 100 genes were specifically expressed in neo-tetraploid rice or autotetraploid rice, and 596, 644 and 565 specific PAVs in neo-tetraploid rice lines influenced the length of chromosome in varying degrees. These results indicated that chromosome breakage, illegitimate recombination and genome rearrangement have altered neo-tetraploid rice genomic structure, which affect transcriptome, proteins and even phenotype. Meanwhile, a large number of SNPs and InDels in neo-tetraploid rice increased genomic polymorphisms. It might be resulted in some homologous chromosome failed to pair together during meiosis, which may reduce homologous recombination rates. It is clear that greater chiasma and multivalent frequency cause low fertility in neo-autopolyploid, and high levels of aneuploidy associated with high numbers of multivalent at metaphase I [56, 57]. Hence, low multivalent frequency may associate with high fertility in polyploids. Multivalent frequency in neo-tetraploid rice was significantly lower than autotetraploid, while bivalent frequency was significantly higher in neo-tetraploid rice than autotetraploid. We inferred that genomic structural reprogramming may lead to high fertility in neo-tetraploid rice.

### Epigenetic reprogramming may associates with fertility in neo-tetraploid rice

Epigenetics plays a crucial role in various aspects of plant biology, including development, silencing of transposable elements and maintenance of genome stability. In plants, epigenetic regulation involves histone and DNA modifications, and ncRNA [58]. Inter-species hybridization in rice has been shown to be associated with changes in the expression levels of genes involved in epigenetic mechanisms [59]. Neo-tetraploid rice is an intersubspecific hybrid (*indica* × *japonica*) of autotetraploid rice, so we inferred epigenetic in neo-tetraploid rice have reprogrammed. In fact, transposon elements and ncRNA displayed specific or differential expressions in neo-tetraploid rice or autotetraploid rice, which showed epigenetic changes in neo-tetraploid rice. Transposon elements are the target of small interfering RNA mediated silencing [60]. Some studies suggested that siRNAs regulate the genes expression and play a crucial role in male meiosis and pollen development [2,20]. Many ncRNAs are functional and involved in regulating genes expression at the transcriptional and post-transcriptional level [58]. So expression levels of many genes may change under the influence of ncRNA and TEs-siRNAs-triggered methylation, particularly during the crucial stage of meiosis, which lead to high fertility in neo-tetraploid rice.

## Supporting information

S1 File Additional figures about distribution and annotation of SNPs and InDels in this study. Frequency of substitution types in SNPs and length distribution of InDels in Neo-tetraploid rice lines vs autotetraploid rice lines (T44 & T45) (Fig A). Annotation of SNPs and InDels between autotetraploid rice and Neo-tetraploid rice lines (Fig B, Fig C and Fig D).
(PDF)

S1 Table Primers used for PCR amplification. (XLSX)

S2 Table Primers used for qRT-PCR. (XLSX)

S3 Table The seed setting of hybrid lines generated by crossing with various autotetraploid lines. (XLSX)

S4 Table Summary of sequencing and mapping results of neo-tetraploid rice lines and their autotetraploid parents. (XLSX)

S5 Table The total number of DNA polymorphisms in neo-tetraploid rice lines and its autotetraploid parents compared with reference genome. (XLSX)

S6 Table Total number of SNPs and InDels in three neo-tetraploid lines and autotetraploid parents. (XLSX)

S7 Table Validation of re-sequencing variations by Sanger sequencing. (XLSX)

S8 Table The number and frequency of SNPs on the rice chromosomes. (XLSX)

S9 Table The number and frequency of InDels on the rice chromosomes. (XLSX)

S10 Table The number of presence/absence variations (PAVs) of various sizes discovered by next-generation genome sequencing in neo-tetraploid rice. (XLSX)

S11 Table Non-synonymous variations in neo-tetraploid rice compared to parents. (XLSX)

S12 Table The list of large-effect SNP and/or InDel genes. (XLSX)

S13 Table The specific variations in neo-tetraploid rice. (XLSX)

S14 Table GO analysis of peculiar mutated genes in neo-tetraploid rice. (XLSX)

S15 Table Meiosis-related genes with peculiar variations in neo-tetraploid rice. (XLSX)

S16 Table The differentially expressed genes in neo-tetraploid rice compared to T44. (XLSX)

S17 Table The differentially expressed genes in neo-tetraploid rice lines compared to T45. (XLSX)

S18 Table Differentially expressed ncRNA in neo-tetraploid rice compared to T44. (XLSX)

S19 Table Differentially expressed transposon elements (TEs) in neo-tetraploid rice compared to T44. (XLSX)

S20 Table The Specific expressed genes in neo-tetraploid rice lines and T44. (XLSX)

S21 Table The specific mutative and differentially expressed genes in neo-tetraploid rice lines compared to T44. (XLSX)

S22 Table The differentially expressed transposons element (TEs) in neo-tetraploid rice compared to T45. (XLSX)

S23 Table The differentially expressed ncRNA in neo-tetraploid rice compared to T45. (XLSX)

S24 Table The specific expressed genes in neo-tetraploid rice lines and T45. (XLSX)

S25 Table The specific mutative and differentially expressed genes in neo-tetraploid rice compared to Jackson-4x (T45). (XLSX)

S26 Table The differentially expressed fertility related genes with specific mutations in neo-tetraploid rice compared to parents. (XLSX)

## Abbreviations

PAVs: presence/absence variations
DEGs: differentially expressed genes
InDels: insertion–deletion polymorphisms
SNPs: single nucleotide polymorphisms
TEs: transposable elements
