# Supplementary material for: Re-sequencing and transcriptome analysis reveal rich DNA variations and differential expressions of fertility-related genes in neo-tetraploid rice
Source: PLoS One. 2019 Apr 5;14(4):e0214953. doi: 10.1371/journal.pone.0214953 (PMC6450637; doi:10.1371/journal.pone.0214953)
Supplement: S1 File — Frequency of substitution types in SNPs and length distribution of InDels in Neo-tetraploid rice lines vs autotetraploid rice lines (T44 & T45) (Fig A). Annotation of SNPs and InDels between autotetraploid rice and Neo-tetraploid rice lines (Fig B, Fig C and Fig D). (PDF) [file pone.0214953.s001.pdf]

Supporting Information

S1 File. Additional figures about distribution and annotation of SNPs and InDels in this study.

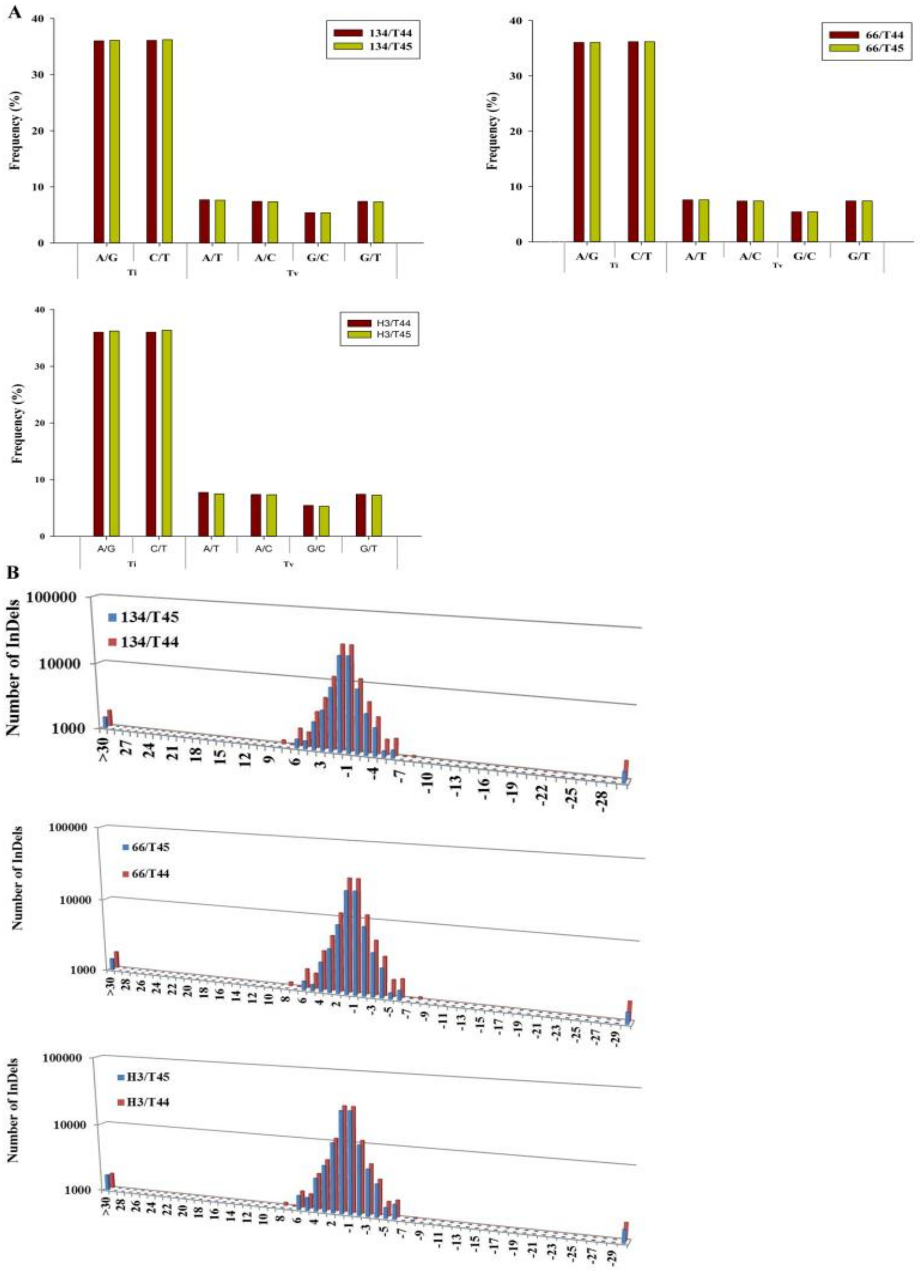

Fig A. Frequency of SNPs substitutions and length distribution of InDels in Neo-tetraploid rice lines compared to their parents.

(A) Frequency of SNPs substitutions in Neo-tetraploid rice lines compared to T45 and T44. (B) Length distribution of InDels. Number of insertions and deletions (y-axis) of various lengths (x-axis, in bp) are shown in the bar graph. Neo-tetraploid rice lines/T44 indicates InDels identified between neo-tetraploid rice and its autotetraploid parent (T44), neo-tetraploid rice lines/T45 indicates InDels identified between neo-tetraploid rice and its autotetraploid parent (T45).

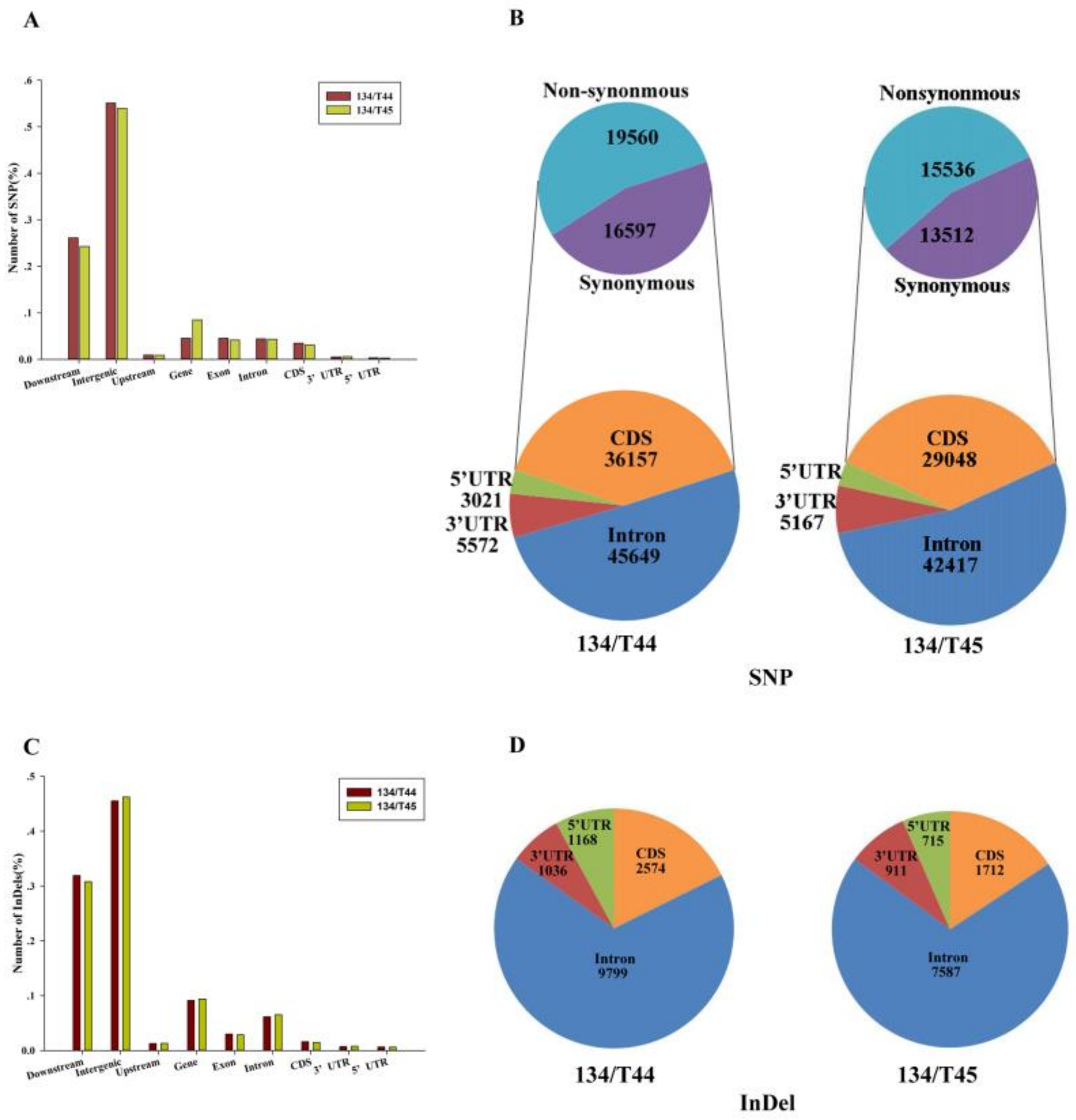

Fig B. Annotation of SNPs and InDels.

Distribution of SNPs (A) and InDels (C) in different genomic regions of 134/T44 and 134/T45. (B, D) Distribution of SNPs (B) and InDels (D) in different genic regions. The number of synonymous and nonsynonymous SNPs detected within the CDS region.

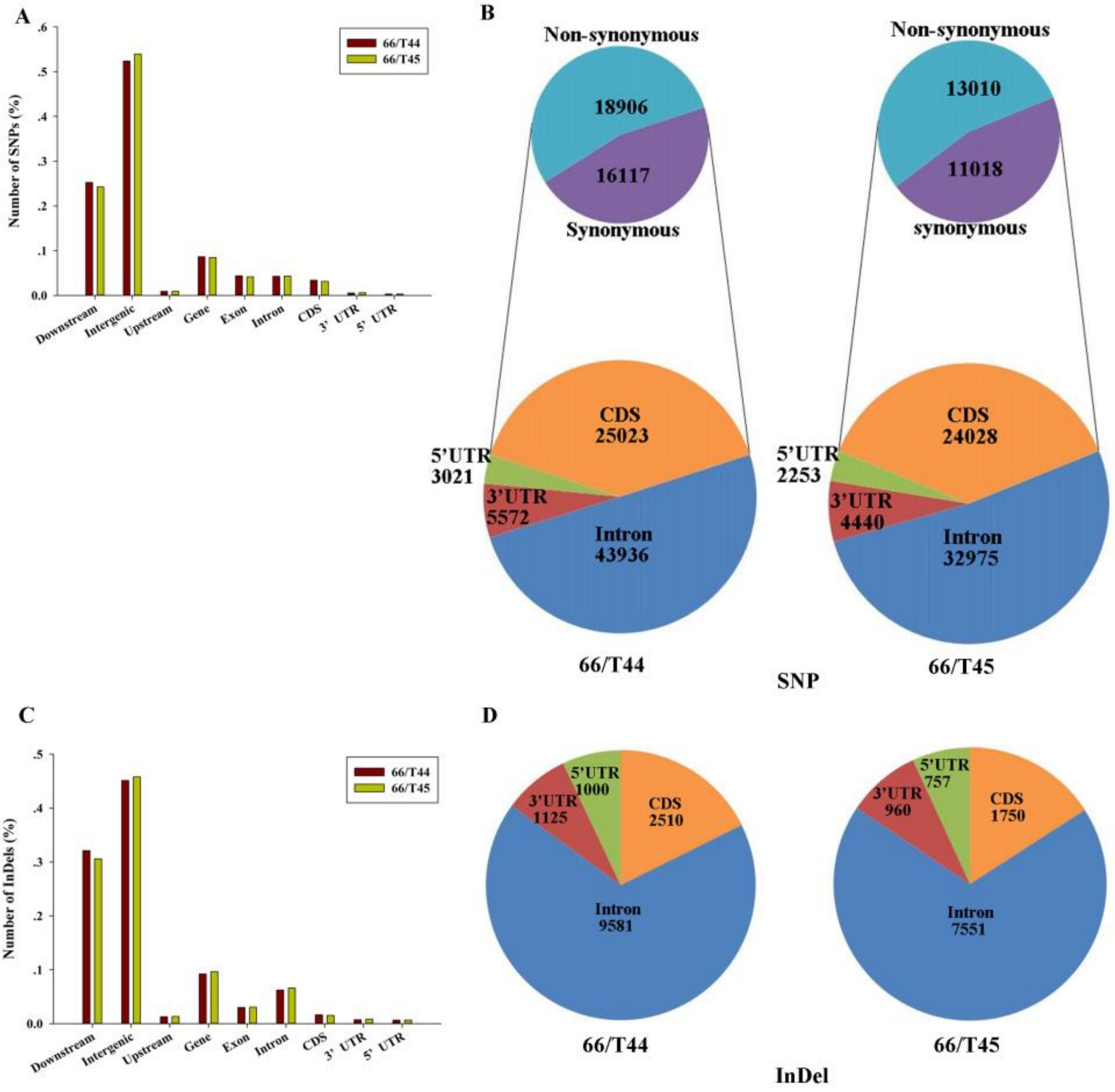

Fig C. Annotation of SNPs and InDels.

Distribution of SNPs (A) and InDels (C) in different genomic regions of 66/T44 and 66/T45. (B, D) Distribution of SNPs (B) and InDels (D) in different genic regions. The number of synonymous and nonsynonymous SNPs detected within the CDS region.

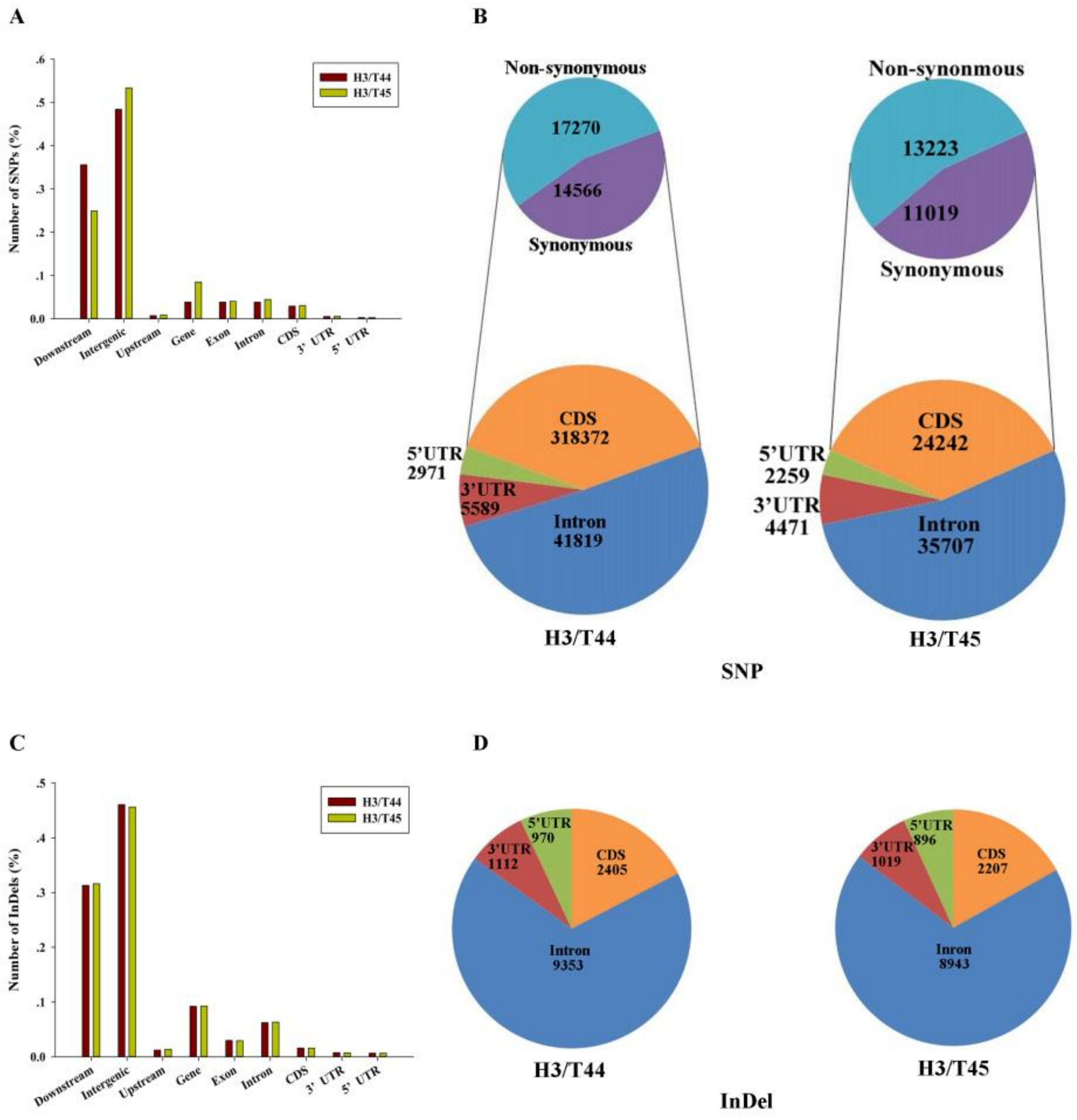

Fig D. Annotation of SNPs and InDels.

Distribution of SNPs (A) and InDels (C) in different genomic regions of H3/T44 and H3/T45. (B, D) Distribution of SNPs (B) and InDels (D) in different genic regions. The number of synonymous and nonsynonymous SNPs detected within the CDS region.
